# *PAX6* gene analysis in irido-fundal coloboma

**Published:** 2011-05-27

**Authors:** Kishlay Kumar, Mukesh Tanwar, Prashant Naithani, Rajpal Insaan, Satpal Garg, Pradeep Venkatesh, Rima Dada

**Affiliations:** 1 Laboratory For Molecular Reproduction and Genetics, Department of Anatomy, All India Institute of Medical Sciences, Ansari Nagar, New Delhi, India; 2 Dr. R. P. Centre for Ophthalmic Sciences, All India Institute of Medical Sciences, Ansari Nagar, New Delhi, India

## Abstract

**Purpose:**

To screen the paired box gene 6 (*PAX6*) gene in irido-fundal coloboma.

**Methods:**

The entire coding region of *PAX6* including intron-exon boundaries was amplified from cases (n=30) and controls (n=30). All sequences were analyzed against the ensemble sequence (ENSG00000007372) for *PAX6*.

**Results:**

DNA sequence analysis of patients and controls revealed a total of three nucleotide changes (g.31815391Cytosine>Thymine; Glycine72Glycine and g.31812215Thymine>Guanine) of which one was neutral/synonymous change and the remaining two were intronic changes. Of these 3 changes, 2 were novel and one was already reported change. All these changes were non-pathogenic, according to in silico analysis.

**Conclusions:**

In our study no pathogenic *PAX6* mutation were identified. This suggests involvement of other coloboma genes. This study expands the SNP spectrum of *PAX6*, only rare variations which are not causative have been found. Since this is a pilot study in the north Indian population, results should be confirmed in different populations by similar studies. Familial cases are required for determining the underlying genetic loci accounting for this clinical phenotype and may lead to better understanding of disease pathogenesis.

## Introduction

**Figure 1:**
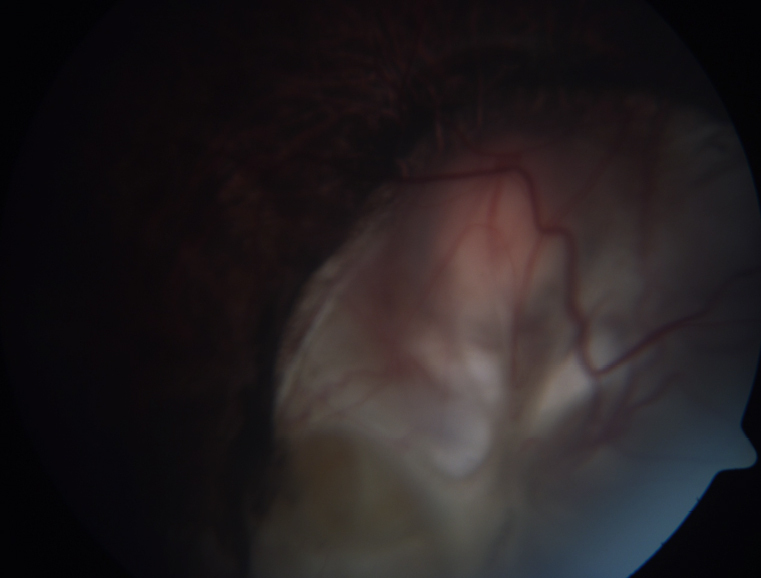
Fundus photograph in type I coloboma.

**Figure 2:**
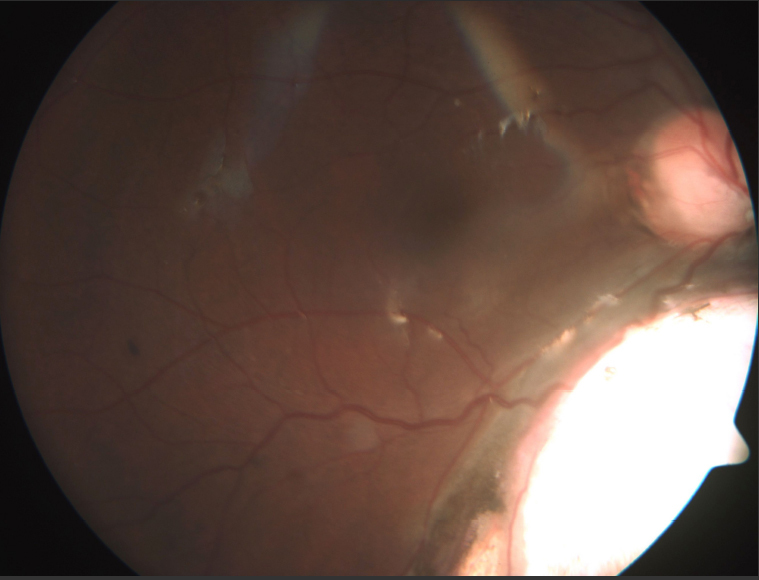
Fundus photograph in type III coloboma.

**Figure 3:**
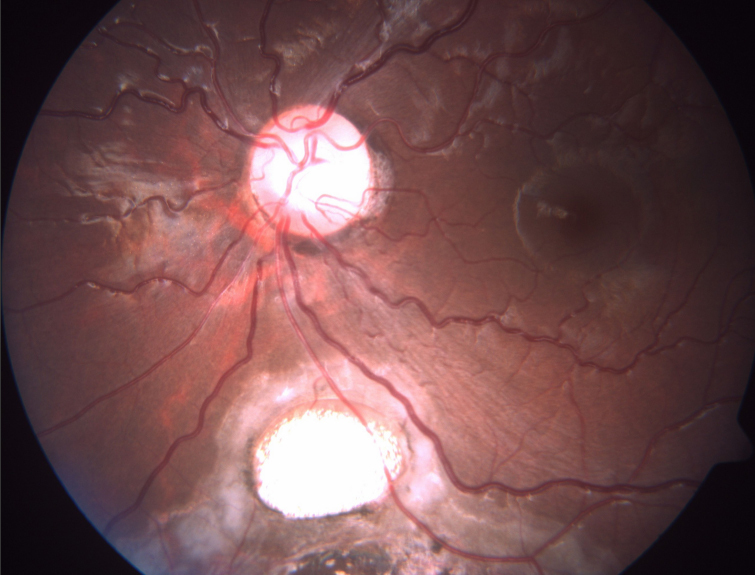
Fundus photograph in type V coloboma.

Congenital malformations of the eyeball appear to occur more frequently in certain countries like India and Sri Lanka, accounting for approximately 25% of cases of visual impairment and blindness [1]. Ocular coloboma is a congenital, common, and heterogeneous malformation which includes a spectrum of anomalies ranging from iris coloboma to clinical anophthalmos. Ocular coloboma, as an isolated defect, is usually inherited as an autosomal dominant disorder, although autosomal recessive inheritance also occurs. Coloboma is a hole in one of the structures of the eye, such as the iris, retina, choroid, or optic disc. If coloboma is present both in iris and retina it is known as irido-fundal coloboma. According to coloboma location in the retina and whether it involves the optic nerve or not, coloboma is classified into six types by Gopal [2]: (I) this involves the optic nerve and is the biggest type of coloboma (Figure 1). Vision is generally quite poor in this type; (II) involves the optic nerve but it is smaller than type I, so vision is poor but not as bad as in type I coloboma; (III) this just misses the optic nerve (Figure 2) so vision can be quite good. However if associated with increased axial length and resulting high myopic refractive error (spectacle number) the vision can be decreased; (IV) this coloboma involves only the optic nerve but not much retina that surrounds it. Vision can be good to average depending on how much the optic nerve is distorted; (V) this coloboma is in the periphery and does not involve the optic nerve as well as the main area of the retina (Figure 3). As far as vision is considered it is quite good in this type and any problem in vision might not be noticed by the patient; (VI) these are partially aborted attempts at forming coloboma. Vision is unaffected in this type of coloboma.

The estimated prevalence of coloboma is 1 in 10,000 in the world population [3,4]. Coloboma associated with other syndromes may cause up to 10% of the childhood blindness [5]. The genetic basis of coloboma remains elusive. Recent studies suggest that earlier developmental processes in eye are controlled by a complex network of transcriptional factors, cell cycle regulators and diffusible signaling molecules [6].

Paired box gene 6 (*PAX6*), a member of the paired box family of transcription factors, has been identified as a key regulator of eye development in both vertebrates and invertebrates [7-9]. During early mouse eye development, expression of Pax6 has been shown in the presumptive lens ectoderm, lens placode, lens vesicle and optic vesicle [10,11]. At later stages, Pax6 is found in epithelia of conjunctiva, cornea, lens, and ciliary body and in the neural retina [12,13]. In humans, mutations in *PAX6* have been demonstrated in several patients with aniridia [14-17], a panocular disease that is associated with iris hypoplasia, corneal opacification, cataract, foveal dysplasia, and other diseases [18].

*PAX6* is located on chromosome 11p13 acts as transcriptional regulator of other genes that are associated with coloboma. This gene contains 14 exons and encodes a 422-amino acid polypeptide containing two DNA-binding domains, a bipartite paired domain, and a paired type homeodomain [19]. The paired domain, which is coded by exons 5–7 of *PAX6*, has two subdomains: the relatively conserved 74-amino acid NH_2_-terminal sub-domain and the more divergent 54-amino acid COOH-terminal subdomain. The latter sub-domain is a common place for mutations [19,20]. Currently there are around 500 mutations that have been reported (Human PAX6 Allelic Variant Database (HPAVD). Most *PAX6* nonsense mutations lead to aniridia, while missense mutations are related to foveal hypoplasia, congenital cataracts, or anterior segment anomalies [21,22].

In this preliminary pilot study we have screened *PAX6* for mutations in irido-fundal coloboma patients (n=30) and normal healthy controls (n=30).

## Methods

### Patient selection and DNA isolation

The research followed the tenets of the Declaration of Helsinki in the treatment of the subject reported herein. The study was approved by institutional review board (IRB # IRB00006862) of All India Institute of Medical Sciences (AIIMS) and all participants gave their written informed consent. A total of thirty coloboma patients presented at the Dr. R. P. Centre for Ophthalmic Sciences (AIIMS, New Delhi, India) were enrolled in this study. Clinical evaluation involved fundoscopy (direct and indirect ophthalmoscopy), slitlamp-biomicroscopy, and retinoscopy. Of these patients, 18 were males and 12 were females. Mean age of presentation was 16.32 years. Diagnosis of coloboma involved the presence of deficient of iris tissue and presence of coloboma in retina on clinical examination. All cases were sporadic without any family history. All cases secondary to causes like trauma etc. were excluded from the study.

After informed consent, detailed personal, medical, and occupational history was collected and a family tree up to three generations was drawn. Thirty ethnically matched normal individuals without any ocular disorder were enrolled as controls. Health information was obtained from controls through the questionnaire; all underwent ophthalmological examination and a blood sample (5 ml) was collected in EDTA (EDTA) vacutainers (Greiner Bio-One, GmbH, Frickenhausen, German**y**) from patients and controls for DNA extraction. DNA was extracted from whole blood samples of all patients and controls using the phenol-chloroform method.

### Polymerase chain reaction (PCR) and DNA Sequencing

All coding regions of *PAX6* including exon-intron junctions were amplified using a set of eight oligonucleotide primers (Table 1). These primers were designed using NCBI PRIMER3 program.

**Table 1:** Primers used for *PAX6* amplification.

**Sample number**	**Forward primer sequence (5′-3′)**	**Reverse primer sequence (5′-3′)**	**Product size (bp)**	**Annealing temp (°C)**
1	GGAGTTCAGGCCTACCTGATGCAG	GAGAAGAGCCAAGCAAACGCCCTC	313	55
2	CGCCGAGGTTGGCACAGGTT	AGCATGGGCTGGGGAGAGCA	710	55
3	CGTTTTGATGCATCTTCAGGCAGTG	GGGCATTCCTCTCTGTTCCCCCA	703	62
4	TGGTGAAGGACCCCCTCCGC	ACACACACGCACCCACCAGC	647	61
5	ACCAGGCCCCTTTTGGAGGCT	TGGCATTCAGTGACCTTTCTGTGGC	554	62
6	GGAGTGGGGAGGTGGGAACCA	AGGCCCTGAGCCACTCCTCAC	837	60
7	AGCTGTGGCCAGTGGAAGGAC	TGGGCCCCCTACTGAGCTTCG	636	62
8	TCCTTTGGATTGGGGTGGGGG	CGTGGCAAAGCTTGTTGATCATGG	713	62

Each reaction was performed in a 25 µl mixture containing 0.2 µM each primer, 0.5 U Taq DNA polymerase, 2.5 µl of 10× PCR buffer (Biogene, New Delhi, India) with 2.5 mM MgCl_2_, and approximately 100 ng genomic DNA. PCR was performed in thermal cycler (My Cycler; BioRad, Gurgaon, India) under the conditions shown in Table 1.

All PCR products were analyzed on 1.8% agarose gel stained with ethidium-bromide (EtBr; 10 mg/ml). Agarose gels were analyzed using the Gel Documentation System (Applied Biosystems, Carlsbad, CA). Amplified PCR products were purified using gel/PCR DNA fragments extraction kit (Catalog number DF100; Geneaid Biotech Ltd., Sijhih City, Taiwan). Purified PCR products were outsourced for sequencing at Molecular Cloning Laboratories (MCLAB, South San Francisco, CA).

DNA sequences were compared with the human PAX6 reference sequence.

### Insilico analysis for predicting pathogenicity of mutations

An improved splice site predictor tool [23] was used for predicting the effect of an intronic nucleotide change on splicing of *PAX6* mRNA.

## Results

DNA sequence analysis of patients and controls revealed a total of three nucleotide changes. Of which one was neutral/synonymous and novel change. The remaining two changes were intronic, one of which was novel. Details of these cases are tabulated (Table 2).

**Table 2:** Clinical manifestations of cases with irido-fundal coloboma.

**Patient ID**	**Age (years)**	**Sex**	**Iridofundla coloboma type**	**Axial length (RE;LE)**	**Visual acuity (RE;LE)**	**Treatment**
IFC1	15	M	B/L Type II IFC	21.62;21.59	3/60; 3/60	B/L laser
IFC2	7	F	B/LType I IFC + microophthalmia +microcornea + nystagamus	24.26;25.50	2/60; 1/60	Laser not possible due to extreme microcornea
IFC3	17	F	LE Inferonasal + IFC type III	20.64;20.33	6/6; PL +ve	LE laser
IFC4	7	M	B/L IFC type I	19.25;22.20	PL+; PL +ve	No laser done
IFC5	10	F	B/L microophthalmia +microcornea + nystagamus + type II IFC	20.21;20.85	1/60;1/60	No laser done due to extreme microcornea
IFC6	27	M	B/L Nystagymus + type II IFC	26.22; 22.71	FCCF;FCCF	B/L laser
IFC7	13	F	RE IFC type II + amblyopia	24.81;22.69	FCCF;6/12	RE laser
IFC8	28	M	LE type III IFC	22.84;21.77	6/6; 6/60	L/E laser
IFC9	26	M	RE IFC type III	26.11;22.60	6/24; 6/6	RE laser
IFC10	16	F	B/L IFC type I	24.50;24.60	1/60;2/60	B/L laser
IFC11	10	F	RE IFC type III	21.54;20.70	Patient non-cooperative	RE laser
IFC12	7	M	RE IFC; L Iris coloboma; B/L microcornea	20.01; 20.39	FCCF;6/60	B/L laser
IFC13	3	M	B/L (microophthalmia +microcornea + distichiasis + IFC type I	20.08;22.00	FCCF;1/60	B/L laser
IFC14	20	M	RE IFC type II coloboma	25.25;23.50	HMCF/6/24	R/L laser
IFC15	5	M	B/L type II IFC	21.91/21.02	6/60; 6/36	B/L laser
IFC16	11	M	B/L microophthalmia + type II IFC	22.65;21.80	3/60;6/60	B/L laser
IFC17	11	M	B/L type I IFC with squint	18.25; 22.45	1/60; 2/60	B/L laser
IFC18	9	M	B/L microophthalmia + IFC type I	22.45; 23.10	PL+; 1/60	B/L laser
IFC19	19	F	B/L microcornea + type II IFC	22.12; 22.70	1/60; 3/60	B/L laser
IFC20	28	F	B/L IFC type III + nystagamus	26.18; 27.66	HMCF/6/60	B/L laser
IFC21	15	M	LE IFC type II	22.73; 26.73	6/6; HMCF	L laser
IFC22	33	M	B/L IFC type III	21.35; 21.50	6/36; 6/36	B/L laser
IFC23	33	M	B/L IFC type IV	24.09; 24.24	6/60; 1/60	B/L laser
IFC24	30	M	B/L IFC type IV + microcornea + nystagymous	29.55; 24.99	HMCF; FC2M	B/L laser
IFC25	25	M	RE type I IFC; LE type V IFC	26.81; 23.71	HMCF/6/6	B/L laser
IFC26	17	M	B/L type III IFC	24.35; 24.50	6/18, 6/18	B/L laser
IFC27	14	M	B/L IFC Type I	26.25; 25.90	1/60; 2/60	B/L laser
IFC28	15	M	LE type III IFC + Microcornea	22.50; 22.24	6/6;6/60	LE laser
IFC29	6	F	RE IFC type III; LE type II IFC	24.45; 25.50	6/60; 2/60	B/L laser
IFC30	12	M	B/L type V IFC	23.50;22.80	6/9; 6/12	B/L laser

Key: IFC represents Iridofundal coloboma; LE represents Left eye; RE represents Right eye; FCCF represents Finger counting close to face; HMCF represents Hand motion close to face; PL represents Perception of light.

### g.31815391Cytosine>Thymine

**Figure 4:**
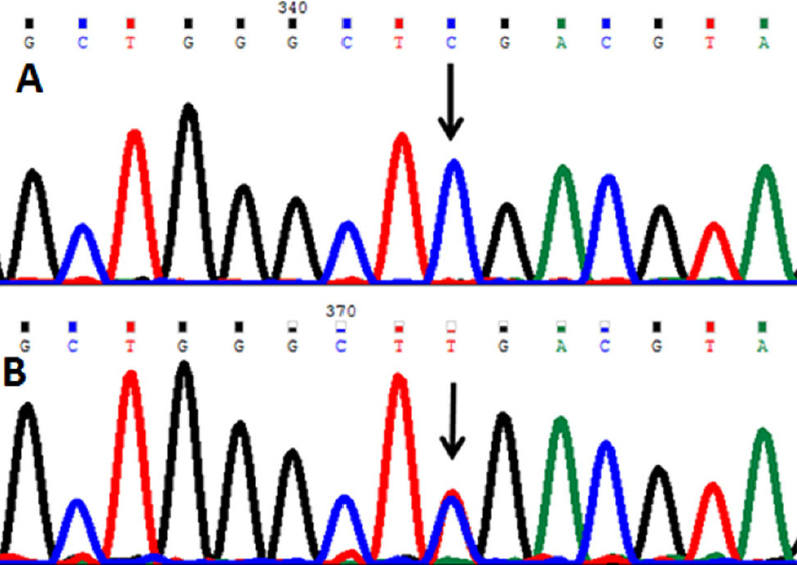
DNA sequence of *PAX6* equivalent to g.31815399 to 31815385. **A**: The reference sequence derived from the control is shown. **B**: The sequence derived from patient shows a heterozygous C>T mutation at g.31815391.

A novel single nucleotide change from cytosine (C) to thymine (T) at genomic position g. 31815391 (Figure 4) was present in one case but absent in controls. The alteration is located in intron 9 (IVS9+40). This change was registered at GenBank with accession number HQ397715.

### g.31823250 Thymine>Guanine

**Figure 5:**
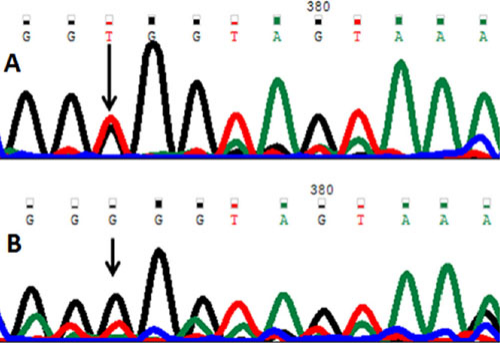
DNA sequence of *PAX6* equivalent to codon 71–75. **A**: The reference sequence derived from the control shows the heterozygous c.216T>G change which predicts a codon change from GGT>GGG and p.G72G mutation. **B**: The sequence derived from another patient shows a homozygous p.G72G mutation.

In this mutation a single nucleotide T was replaced by guanine (G) at genomic position g.31823250; cDNA position c.216; codon 72 resulted in a codon change GGT>GGG which predicts a synonymous change p.Gly72Gly (p.G72G; Figure 5). This change was present as heterozygous change in 29 cases and 20 controls; and as a homozygous change in one case. This change was novel and registered at GenBank with accession number HQ397714.

### g.31812215Thymine>Guanine

**Figure 6:**
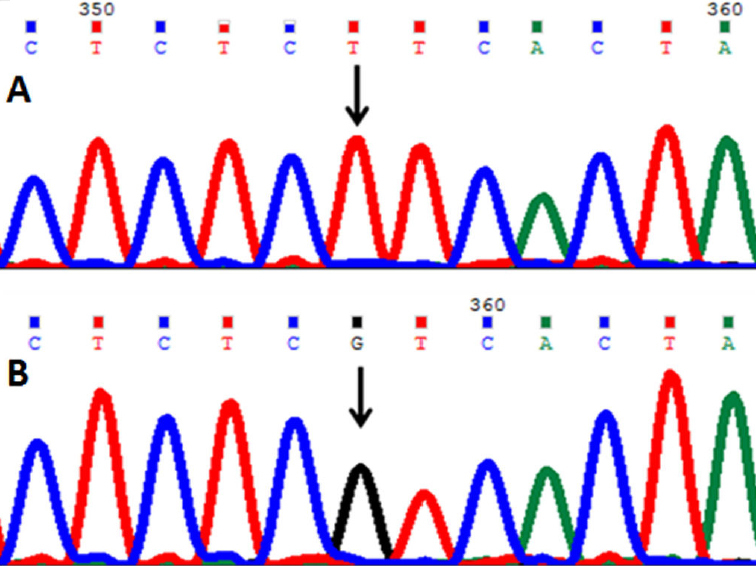
DNA sequence of *PAX6* equivalent to g.31812220 to 31812209. **A**: The reference sequence derived from the control is shown. **B**: The sequence derived from patient shows a homozygous g.31812215T>G mutation.

A single nucleotide change from T to G at genomic position g.31812215 (Figure 6) was present in six cases and one control. The alteration is located in intron 12 (IVS13–42).

Improved splice site prediction for both intronic changes showed that the location of these changes is not present at a splice site and may not create splicing error in the PAX6 protein.

## Discussion

The genetic basis of coloboma remains elusive. Recent studies suggest that earlier developmental process in the eye are controlled by a complex network of transcriptional factors, cell cycle regulators, and diffusible signaling molecules [6]. Mutations in these genes may lead to ocular coloboma. It has been proposed that PAX6 acts as a transcriptional regulator of many other genes involved in ocular development. *PAX6* mutations have been identified in sporadic aniridia cases from different populations [17] as well as in familial aniridia cases [14-16]. In this study we have screened *PAX6* in irido-fundal coloboma patients and controls and we observed 3 changes (2 intronic changes and one synonymous/neutral change). However, no pathogenic *PAX6* mutation was identified in our patients.

Mutations in *PAX2* were identified in individuals with ocular coloboma in renal coloboma syndrome [24]. *PAX2* is a member of a multi-gene family containing a paired box domain that was identical in *Drosophila* and subsequently in vertebrates [25]. *PAX2* expression is critical for development of urogenital tract, central nervous system (CNS), inner ear, and optic nerve. Further studies suggest that *PAX2* mutations are not common in patients with isolated ocular coloboma and associated anomalies [26].

The absence of *PAX6* mutations in our study population suggests the involvement of other coloboma genes showed by Zhang et al. [27]. This study expands the SNP spectrum of *PAX6*, only rare variations which are not causative have been found. Since this is a pilot study in the north Indian population, our results should be confirmed in different populations by similar studies. However, the absence of *PAX6* mutations in selected population do not rule out the possibility of involvement of this gene in coloboma. Friling et al. [28] have shown the deletion of the whole gene (11p) in a patient with uveal colobomata. So there is still a possibility for *PAX6* to be involved in coloboma cases which can be detected by CGH array. As the frequency of the congenital blindness is more in Asian countries [18], where a majority of the population has vitamin A deficiency which is required for normal expression of several genes involved in ocular morphogenesis. Vitamin A is critical during eye development and optic fissure fails to close in vitamin A deficient embryos [29]. It has been reported that *Pitx2* expression is downregulated in vitamin A deficient embryos and this gene is required for optic fissure closure. Epidemiological evidences has shown the teratogenic effect of vitamin A deficiency during pregnancy. Thus screening of the candidate gene is required in large number of cases. Familial cases are required for determination of the underlying genetic loci accounting for this clinical phenotype which may lead to better understanding of disease pathogenesis in near future.

## References

[1] Brémond-Gignac D, Bitoun P, Reis LM, Copin H, Murray JC, Semina EV (2010). Identification of dominant FOXE3 and PAX6 mutations in patients with congenital cataract and aniridia.. Mol Vis.

[2] Gopal L (2008). A clinical and optical coherence tomography study of choroidal colobomas.. Curr Opin Ophthalmol.

[3] Chow RL, Altmann CR, Lang RA, Hemmati-Brivanlou A (1999). Pax6 induces ectopic eyes in a vertebrate.. Development.

[4] Cunliffe HE, McNoe LA, Ward TA, Devriendt K, Brunner HG, Eccles MR (1998). The prevalence of PAX2 mutations in patients with isolated colobomas or colobomas associated with urogenital anomalies.. J Med Genet.

[5] Dandona L, Dandona R, Shamanna BR, Naduvilath TJ, Rao GN (1998). Developing a model to reduce blindness in India: The International Centre for Advancement of Rural Eye Care.. Indian J Ophthalmol.

[6] Davis JA, Reed RR (1996). Role of Olf-1 and Pax-6 transcription factors in neurodevelopment.. J Neurosci.

[7] Epstein J, Cai J, Glaser T, Jepeal L, Maas R (1994). Identification of a Pax paired domain recognition sequence and evidence for DNA-dependent conformational changes.. J Biol Chem.

[8] Gehring WJ, Ikeo K (1999). Pax 6: mastering eye morphogenesis and eye evolution.. Trends Genet.

[9] Grindley JC, Davidson DR, Hill RE (1995). The role of Pax-6 in eye and nasal development.. Development.

[10] Hanson IM (2003). PAX6 and congenital eye malformations.. Pediatr Res.

[11] Jean D, Ewan K, Gruss P (1998). Molecular regulators involved in vertebrate eye development.. Mech Dev.

[12] Kang Y, Yuan HP, Li X, Li QJ, Wu Q, Hu Q (2010). A novel mutation of the PAX6 gene in a Chinese family with aniridia Chinese.. Zhonghua Yi Xue Yi Chuan Xue Za Zhi.

[13] Koroma BM, Yang JM, Sundin OH (1997). The Pax-6 homeobox gene is expressed throughout the corneal and conjunctival epithelia.. Invest Ophthalmol Vis Sci.

[14] Lee H, Khan R, O'Keefe M (2008). Aniridia: current pathology and management.. Acta Ophthalmol.

[15] Marquardt T, Ashery-Padan R, Andrejewski N, Scardigli R, Guillemot F, Gruss P (2001). Pax6 is required for the multipotent state of retinal progenitor cells.. Cell.

[16] Maumenee IH, Mitchell TN (1990). Colobomatous malformations of the eye.. Trans Am Ophthalmol Soc.

[17] Prosser J, van Heyningen V (1998). PAX6 mutations reviewed.. Hum Mutat.

[18] Rahi JS, Sripathi S, Gilbert CE, Foster A (1995). Childhood blindness in India: causes in 1318 blind school students in nine states.. Eye.

[19] Reese MG, Eeckman FH, Kulp D, Haussler D (1997). Improved Splice Site Detection in Genie. J Comput Biol.

[20] Sanyanusin P, McNoe LA, Sullivan MJ, Weaver RG, Eccles MR (1995). Mutation of PAX2 in two siblings with renal-coloboma syndrome.. Hum Mol Genet.

[21] Stoll C, Alembik Y, Dott B, Roth MP (1997). Congenital eye malformations in 212,479 consecutive births.. Ann Genet.

[22] Stoll C, Alembik Y, Dott B, Roth MP (1992). Epidemiology of congenital eye malformations in 131,760 consecutive births.. Ophthalmic Paediatr Genet.

[23] Strachan T, Read AP (1994). PAX genes.. Curr Opin Genet Dev.

[24] Tzoulaki I, White IM, Hanson IM (2005). PAX6 mutations: genotype-phenotype correlations.. BMC Genet.

[25] Walther C, Gruss P (1991). Pax-6, a murine paired box gene, is expressed in the developing CNS.. Development.

[26] Wang LM, Ying M, Wang X, Wang YC, Hao P, Li ND (2009). R240X mutation of the PAX6 gene in a Chinese family with congenital aniridia.. Zhonghua Yi Xue Yi Chuan Xue Za Zhi..

[27] Zhang X, Li S, Xiao X, Jia X, Wang P, Shen H, Guo X, Zhang Q (2009). Mutational screening of 10 genes in Chinese patients with microphthalmia and/or coloboma.. Mol Vis.

[28] Friling R, Yassur Y, Abeliovich D, Biedner B, Galil A, Dagan J (1995). Carmi, R. A complex chromosome translocation resulting in deletion 11p and associated with uveal colobomata.. Ophthalmic Genet.

[29] See AW, Clagett-Dame M (2009). The temporal requirement for vitamin A in the developing eye: mechanism of action in optic fissure closure and new roles for the vitamin in regulating cell proliferation and adhesion in the embryonic retina.. Dev Biol.

